# The genome sequence of the Small Emerald, *Hemistola chrysoprasaria *(Esper, 1795)

**DOI:** 10.12688/wellcomeopenres.19999.1

**Published:** 2023-10-12

**Authors:** Douglas Boyes, John F. Mulley, University of Oxford and Wytham Woods Genome Acquisition Lab, Darwin Tree of Life Barcoding collective, Wellcome Sanger Institute Tree of Life programme, Wellcome Sanger Institute Scientific Operations: DNA Pipelines collective, Tree of Life Core Informatics collective, Darwin Tree of Life Consortium

**Affiliations:** 1 UK Centre for Ecology & Hydrology, Wallingford, England, UK; 2 Bangor University, Bangor, Wales, UK

**Keywords:** Hemistola chrysoprasaria, Small Emerald, genome sequence, chromosomal, Lepidoptera

## Abstract

We present a genome assembly from an individual male *Hemistola chrysoprasaria* (the Small Emerald; Arthropoda; Insecta; Lepidoptera; Geometridae). The genome sequence is 438.2 megabases in span. Most of the assembly is scaffolded into 30 chromosomal pseudomolecules, including the Z sex chromosome. The mitochondrial genome has also been assembled and is 15.63 kilobases in length. Gene annotation of this assembly on Ensembl identified 17,512 protein coding genes.

## Species taxonomy

Eukaryota; Metazoa; Eumetazoa; Bilateria; Protostomia; Ecdysozoa; Panarthropoda; Arthropoda; Mandibulata; Pancrustacea; Hexapoda; Insecta; Dicondylia; Pterygota; Neoptera; Endopterygota; Amphiesmenoptera; Lepidoptera; Glossata; Neolepidoptera; Heteroneura; Ditrysia; Obtectomera; Geometroidea; Geometridae; Geometrinae; *Hemistola*; *Hemistola chrysoprasaria* (Esper, 1795) (NCBI:txid934942).

## Background

The Small Emerald ( *Hemistola chrysoprasaria*) is a geometrid moth with rounded wings (17–20mm forewing length) and white cross-lines on an overall blue-green background. The background colour is bright when newly emerged, but fades to almost white over time. *H. chrysoprasaria* has a Palaearctic distribution, and is widely distributed in the south of England and Wales. There is one generation per year, with a peak flight time of June to August in the UK, and overwintering occurs at the larval stage. Larvae feed primarily on Traveller’s joy ( *Clematis vitalba*), although they may also feed on other species of *Clematis* ( Waring *et al.*, 2017). Transport on cultivated plants may explain sporadic reports of small emeralds outside of the “normal” range (for example, Scotland ( NBN Atlas Partnership, 2023). The conservation status of *H. chrysoprasaria* in Great Britain was assessed as “least concern” in 2019 ( Fox *et al.*, 2019), a potentially encouraging change from “vulnerable” in 2006 ( Conrad *et al.*, 2006; Fox *et al.*, 2006), and “declining” in 2013 ( Fox, 2013).

*H. chrysoprasaria* larvae show an interesting colour change phenomenon, changing from brown during late summer to green in spring, following a period of winter diapause. Such background-matching larval colour change behaviour is known from other species of Lepidoptera, such as the Peppered Moth ( *Biston betularia*), where extraocular photoreception is used to determine background colouration ( Eacock *et al.*, 2017; Eacock *et al.*, 2019). However, the Peppered Moth example seems to be an adaptation to larval dispersal via wind and polyphagy, where larvae can settle on a diverse range of host plants, rather than a temporal change on a single host plant as is the case for *H. chrysoprasaria*. This Small Emerald genome sequence assembly will provide a useful resource for the identification of the molecular basis of this colour change behaviour.

## Genome sequence report

**Figure 1.**
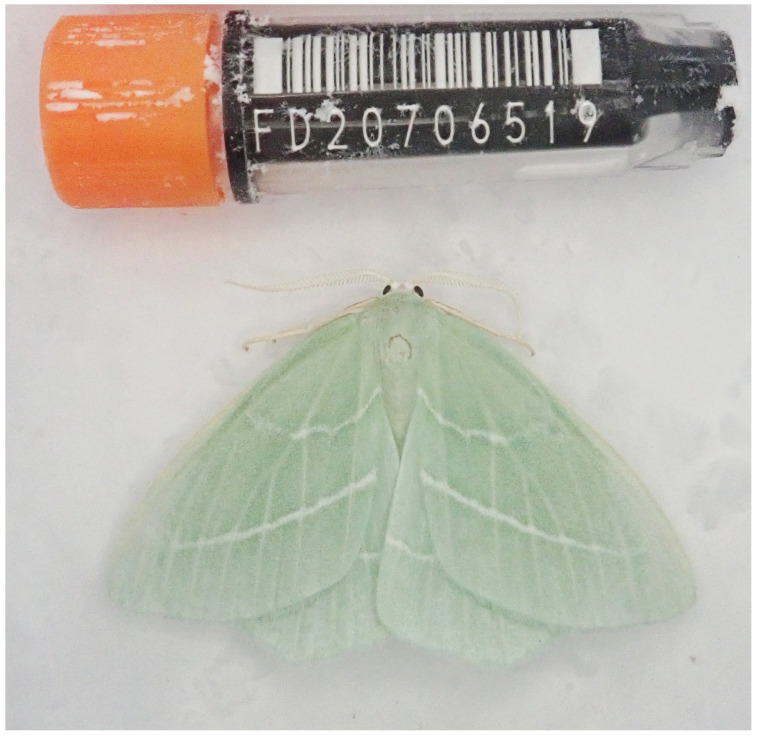
Photograph of the *Hemistola chrysoprasaria* (ilHemChry1) specimen used for genome sequencing.

The genome was sequenced from one male *Hemistola chrysoprasaria* ( Figure 1) collected from Wytham Woods, Oxfordshire, UK (51.77, –1.31). A total of 49-fold coverage in Pacific Biosciences single-molecule HiFi long was generated. Primary assembly contigs were scaffolded with chromosome conformation Hi-C data. Manual assembly curation corrected 17 missing joins or misjoins and removed 3 haplotypic duplications, reducing the scaffold number by 8.57%.

**Figure 2.**
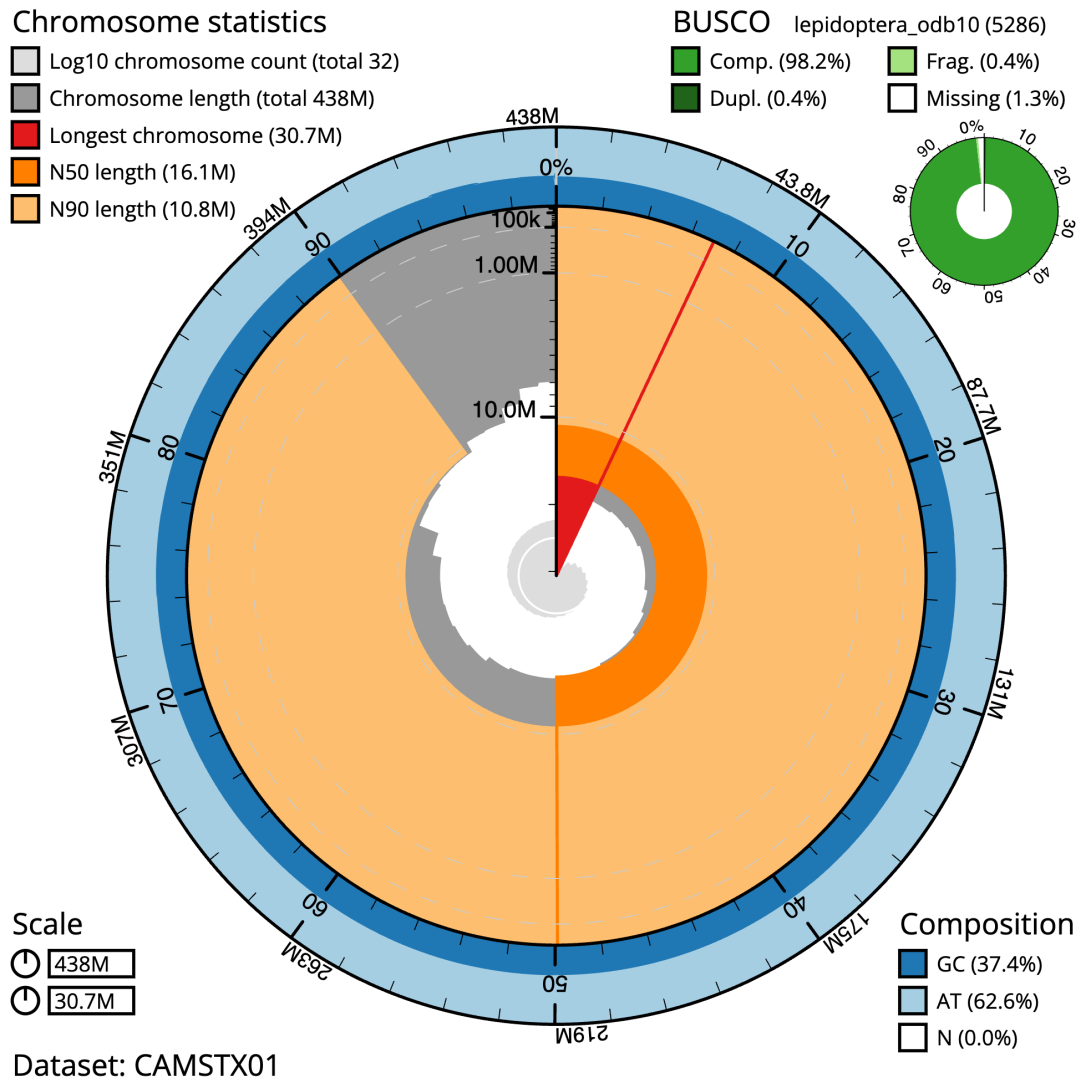
Genome assembly of *Hemistola chrysoprasaria*, ilHemChry1.1: metrics. The BlobToolKit Snailplot shows N50 metrics and BUSCO gene completeness. The main plot is divided into 1,000 size-ordered bins around the circumference with each bin representing 0.1% of the 438,253,172 bp assembly. The distribution of scaffold lengths is shown in dark grey with the plot radius scaled to the longest scaffold present in the assembly (30,684,051 bp, shown in red). Orange and pale-orange arcs show the N50 and N90 scaffold lengths (16,143,413 and 10,750,662 bp), respectively. The pale grey spiral shows the cumulative scaffold count on a log scale with white scale lines showing successive orders of magnitude. The blue and pale-blue area around the outside of the plot shows the distribution of GC, AT and N percentages in the same bins as the inner plot. A summary of complete, fragmented, duplicated and missing BUSCO genes in the lepidoptera_odb10 set is shown in the top right. An interactive version of this figure is available at https://blobtoolkit.genomehubs.org/view/ilHemChry1.1/dataset/CAMSTX01/snail.

The final assembly has a total length of 438.2 Mb in 31 sequence scaffolds with a scaffold N50 of 16.1 Mb ( Table 1). Most (99.98%) of the assembly sequence was assigned to 30 chromosomal-level scaffolds, representing 29 autosomes and the Z sex chromosome. Chromosome-scale scaffolds confirmed by the Hi-C data are named in order of size ( Figure 2– Figure 5; Table 2). While not fully phased, the assembly deposited is of one haplotype. Contigs corresponding to the second haplotype have also been deposited. The mitochondrial genome was also assembled and can be found as a contig within the multifasta file of the genome submission.

**Table 1.** Genome data for *Hemistola chrysoprasaria*, ilHemChry1.1.

Project accession data		
Assembly identifier	ilHemChry1.1	
Species	*Hemistola chrysoprasaria*	
Specimen	ilHemChry1	
NCBI taxonomy ID	934942	
BioProject	PRJEB55573	
BioSample ID	SAMEA10978934	
Isolate information	ilHemChry1, male: abdomen (DNA sequencing); head and thorax (Hi-C sequencing)	
Assembly metrics *		*Benchmark*
Consensus quality (QV)	65	*≥ 50*
*k*-mer completeness	100%	*≥ 95%*
BUSCO **	C:98.2%[S:97.8%,D:0.4%],F:0.4%, M:1.3%,n:5,286	*C ≥ 95%*
Percentage of assembly mapped to chromosomes	99.98%	*≥ 95%*
Sex chromosomes	Z chromosome	*localised homologous pairs*
Organelles	Mitochondrial genome assembled	*complete single alleles*
Raw data accessions		
PacificBiosciences SEQUEL II	ERR10115640	
Hi-C Illumina	ERR10123713	
Genome assembly		
Assembly accession	GCA_947063395.1	
*Accession of alternate haplotype*	GCA_947059775.1	
Span (Mb)	438.2	
Number of contigs	81	
Contig N50 length (Mb)	8.9	
Number of scaffolds	31	
Scaffold N50 length (Mb)	16.1	
Longest scaffold (Mb)	30.7	
Genome annotation		
Number of protein-coding genes	17,512	
Number of gene transcripts	17,669	

* Assembly metric benchmarks are adapted from column VGP-2020 of “Table 1: Proposed standards and metrics for defining genome assembly quality” from ( Rhie *et al.*, 2021). ** BUSCO scores based on the lepidoptera_odb10 BUSCO set using v5.3.2. C = complete [S = single copy, D = duplicated], F = fragmented, M = missing, n = number of orthologues in comparison. A full set of BUSCO scores is available at https://blobtoolkit.genomehubs.org/view/ilHemChry1.1/dataset/CAMSTX01/busco.

**Figure 3.**
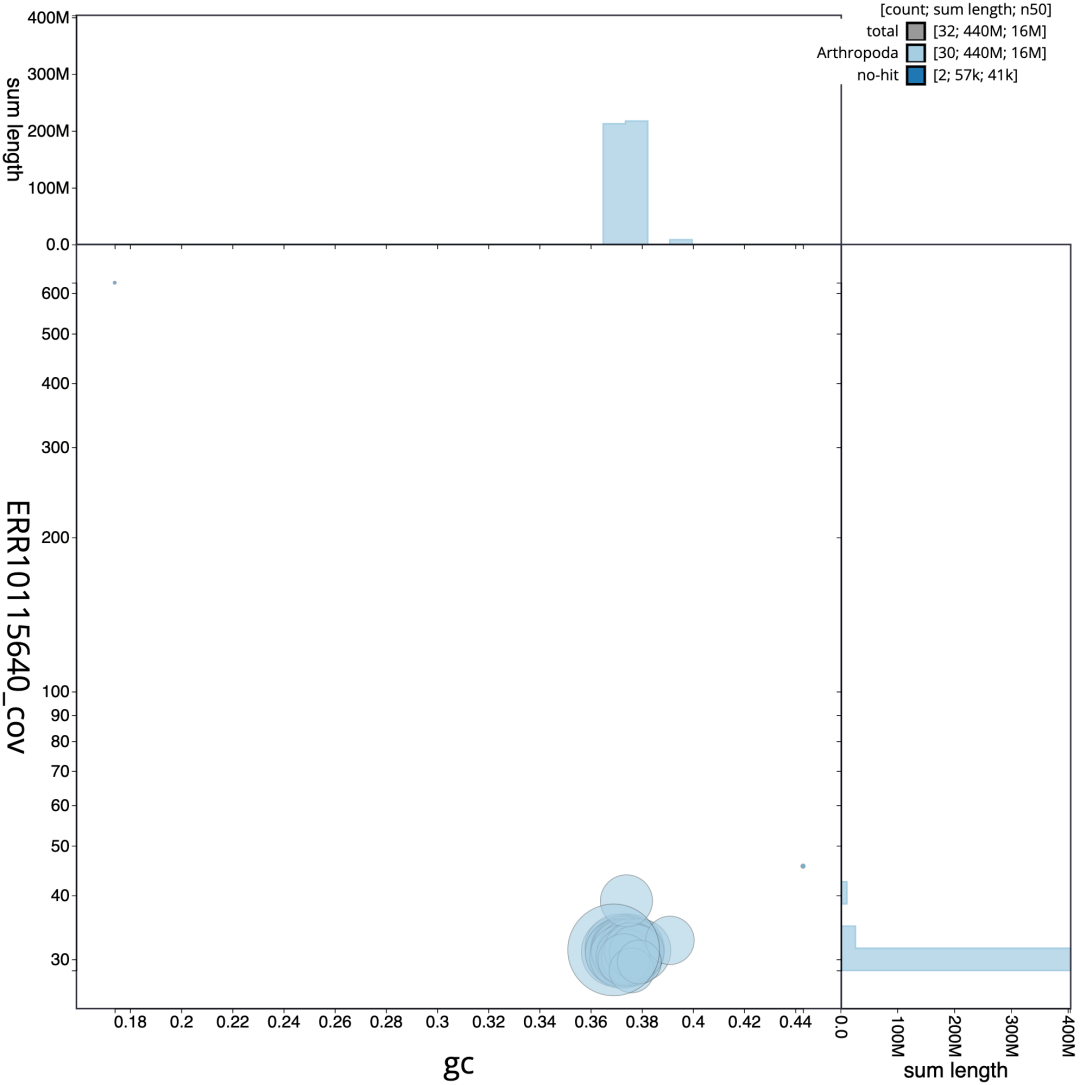
Genome assembly of *Hemistola chrysoprasaria*, ilHemChry1.1: BlobToolKit GC-coverage plot. Scaffolds are coloured by phylum. Circles are sized in proportion to scaffold length. Histograms show the distribution of scaffold length sum along each axis. An interactive version of this figure is available at https://blobtoolkit.genomehubs.org/view/ilHemChry1.1/dataset/CAMSTX01/blob.

**Figure 4.**
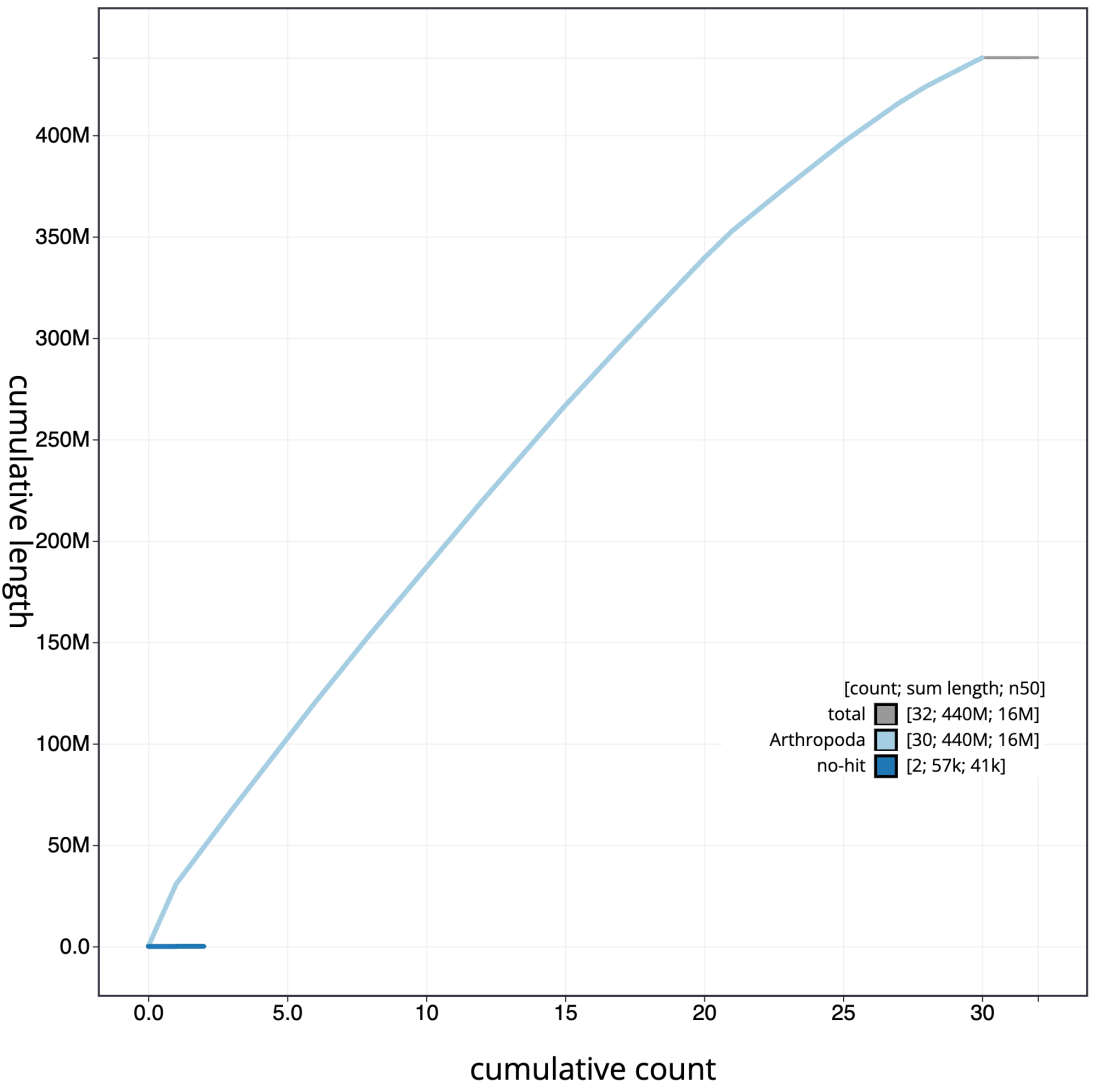
Genome assembly of *Hemistola chrysoprasaria*, ilHemChry1.1: BlobToolKit cumulative sequence plot. The grey line shows cumulative length for all scaffolds. Coloured lines show cumulative lengths of scaffolds assigned to each phylum using the buscogenes taxrule. An interactive version of this figure is available at https://blobtoolkit.genomehubs.org/view/ilHemChry1.1/dataset/CAMSTX01/blob.

**Figure 5.**
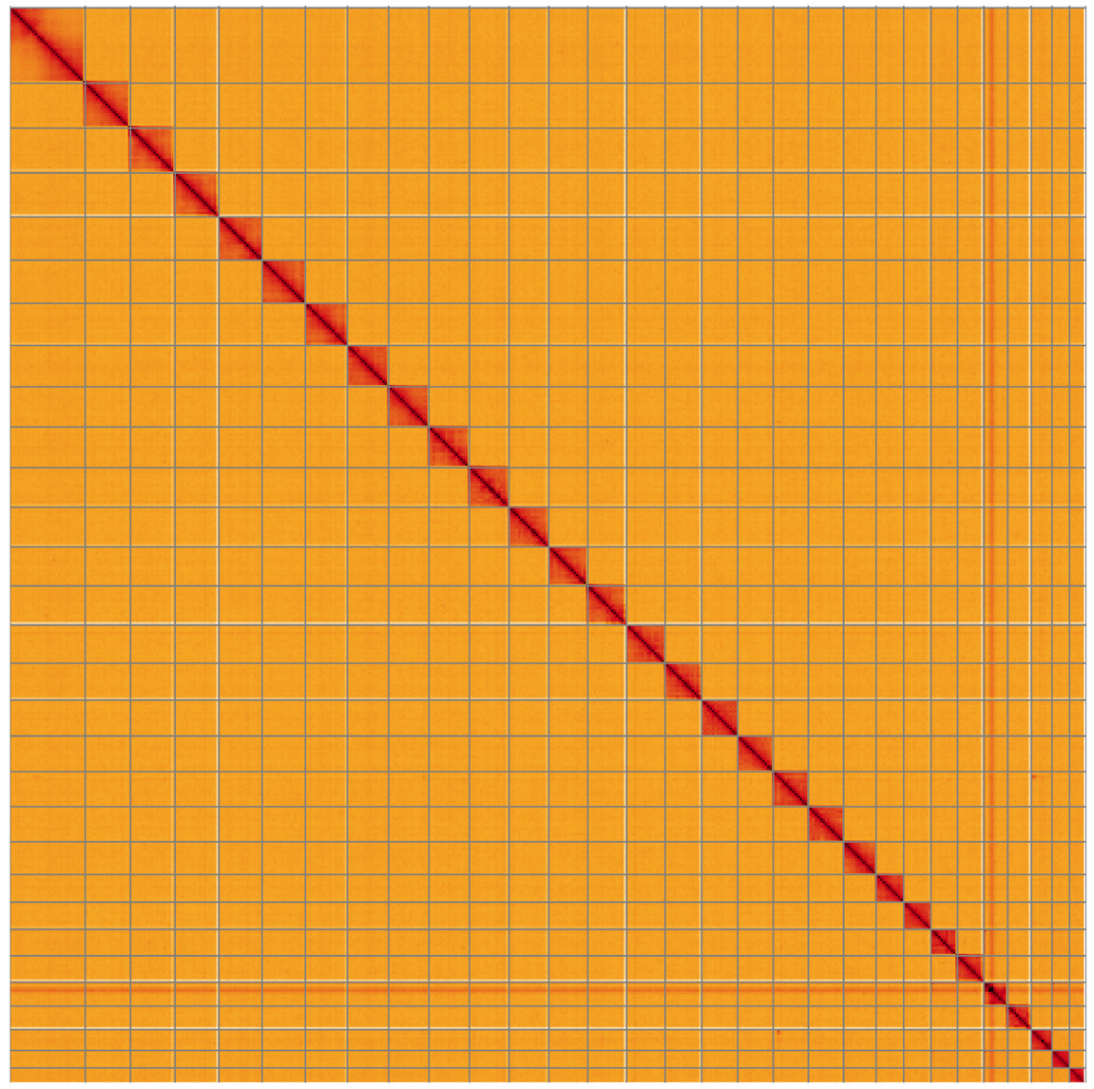
Genome assembly of *Hemistola chrysoprasaria*, ilHemChry1.1: Hi-C contact map of the ilHemChry1.1 assembly, visualised using HiGlass. Chromosomes are shown in order of size from left to right and top to bottom. An interactive version of this figure may be viewed at https://genome-note-higlass.tol.sanger.ac.uk/l/?d=b59mEK0zS3C-ucZrQBgUWg.

**Table 2.** Chromosomal pseudomolecules in the genome assembly of *Hemistola chrysoprasaria*, ilHemChry1.

INSDC accession	Chromosome	Length (Mb)	GC%
OX346710.1	1	18.37	37.5
OX346711.1	2	18.14	37.5
OX346712.1	3	17.89	37.5
OX346713.1	4	17.59	37.5
OX346714.1	5	17.57	37.0
OX346715.1	6	17.15	37.5
OX346716.1	7	16.8	37.0
OX346717.1	8	16.44	37.5
OX346718.1	9	16.43	37.0
OX346719.1	10	16.19	37.0
OX346720.1	11	16.14	37.0
OX346721.1	12	15.87	37.5
OX346722.1	13	15.86	37.5
OX346723.1	14	15.59	37.5
OX346724.1	15	14.94	37.5
OX346725.1	16	14.65	37.5
OX346726.1	17	14.44	37.5
OX346727.1	18	14.33	37.0
OX346728.1	19	14.3	38.0
OX346729.1	20	13.31	37.5
OX346730.1	21	11.17	37.5
OX346731.1	22	11.08	37.5
OX346732.1	23	10.78	37.5
OX346733.1	24	10.75	38.0
OX346734.1	25	9.74	37.5
OX346735.1	26	9.63	37.5
OX346736.1	27	8.35	39.0
OX346737.1	28	7.14	37.5
OX346738.1	29	6.86	38.0
OX346709.1	Z	30.68	37.0
OX346739.1	MT	0.02	17.5

The estimated Quality Value (QV) of the final assembly is 65 with *k*-mer completeness of 100%, and the assembly has a BUSCO v5.3.2 completeness of 98.2% (single = 97.8%, duplicated = 0.4%), using the lepidoptera_odb10 reference set ( *n* = 5,286).

Metadata for specimens, spectral estimates, sequencing runs, contaminants and pre-curation assembly statistics can be found at https://links.tol.sanger.ac.uk/species/934942.

## Genome annotation report

The *Hemistola chrysoprasaria* genome assembly (GCA_947063395.1) was annotated using the Ensembl rapid annotation pipeline ( Table 1; https://rapid.ensembl.org/Hemistola_chrysoprasaria_GCA_947063395.1/Info/Index). The resulting annotation includes 17,669 transcribed mRNAs from 17,512 protein-coding genes.

## Methods

### Sample acquisition and nucleic acid extraction

A male *Hemistola chrysoprasaria* (specimen ID Ox001665, ToLID ilHemChry1) was collected from Wytham Woods, Oxfordshire, UK (latitude 51.77, longitude –1.31) on 2021-07-17, using a light trap. The specimen was collected and identified by Douglas Boyes (University of Oxford) and snap-frozen on dry ice.

DNA was extracted at the Tree of Life laboratory, Wellcome Sanger Institute (WSI). The ilHemChry1 sample was weighed and dissected on dry ice with tissue set aside for Hi-C sequencing. Abdomen tissue was disrupted using a Nippi Powermasher fitted with a BioMasher pestle. High molecular weight (HMW) DNA was extracted using the Qiagen MagAttract HMW DNA extraction kit. HMW DNA was sheared into an average fragment size of 12–20 kb in a Megaruptor 3 system with speed setting 30. Sheared DNA was purified by solid-phase reversible immobilisation using AMPure PB beads with a 1.8X ratio of beads to sample to remove the shorter fragments and concentrate the DNA sample. The concentration of the sheared and purified DNA was assessed using a Nanodrop spectrophotometer and Qubit Fluorometer and Qubit dsDNA High Sensitivity Assay kit. Fragment size distribution was evaluated by running the sample on the FemtoPulse system.

### Sequencing

Pacific Biosciences HiFi circular consensus DNA sequencing libraries were constructed according to the manufacturers’ instructions. DNA sequencing was performed by the Scientific Operations core at the WSI on a Pacific Biosciences SEQUEL II (HiFi) instruments. Hi-C data were also generated from head and thorax tissue of ilHemChry1 using the Arima2 kit and sequenced on the llumina NovaSeq 6000 instrument.

### Genome assembly, curation and evaluation

Assembly was carried out with Hifiasm ( Cheng *et al.*, 2021) and haplotypic duplication was identified and removed with purge_dups ( Guan *et al.*, 2020). The assembly was then scaffolded with Hi-C data ( Rao *et al.*, 2014) using YaHS ( Zhou *et al.*, 2023). The assembly was checked for contamination and corrected as described previously ( Howe *et al.*, 2021). Manual curation was performed using HiGlass ( Kerpedjiev *et al.*, 2018) and Pretext ( Harry, 2022). The mitochondrial genome was assembled using MitoHiFi ( Uliano-Silva *et al.*, 2023), which runs MitoFinder ( Allio *et al.*, 2020) or MITOS ( Bernt *et al.*, 2013) and uses these annotations to select the final mitochondrial contig and to ensure the general quality of the sequence.

A Hi-C map for the final assembly was produced using bwa-mem2 ( Vasimuddin *et al.*, 2019) in the Cooler file format ( Abdennur & Mirny, 2020). To assess the assembly metrics, the *k*-mer completeness and QV consensus quality values were calculated in Merqury ( Rhie *et al.*, 2020). This work was done using Nextflow ( Di Tommaso *et al.*, 2017) DSL2 pipelines “sanger-tol/readmapping” ( Surana *et al.*, 2023a) and “sanger-tol/genomenote” ( Surana *et al.*, 2023b). The genome was analysed within the BlobToolKit environment ( Challis *et al.*, 2020) and BUSCO scores ( Manni *et al.*, 2021; Simão *et al.*, 2015) were calculated.

Table 3 contains a list of relevant software tool versions and sources.

**Table 3.** Software tools: versions and sources.

Software tool	Version	Source
BlobToolKit	4.1.2	https://github.com/blobtoolkit/blobtoolkit
BUSCO	5.3.2	https://gitlab.com/ezlab/busco
Hifiasm	0.16.1-r375	https://github.com/chhylp123/hifiasm
HiGlass	1.11.6	https://github.com/higlass/higlass
Merqury	MerquryFK	https://github.com/thegenemyers/MERQURY.FK
MitoHiFi	2	https://github.com/marcelauliano/MitoHiFi
PretextView	0.2	https://github.com/wtsi-hpag/PretextView
purge_dups	1.2.3	https://github.com/dfguan/purge_dups
sanger-tol/genomenote	v1.0	https://github.com/sanger-tol/genomenote
sanger-tol/readmapping	1.1.0	https://github.com/sanger-tol/readmapping/tree/1.1.0
YaHS	yahs-1.1.91eebc2	https://github.com/c-zhou/yahs

### Genome annotation

The BRAKER2 pipeline ( Brůna *et al.*, 2021) was used in the default protein mode to generate annotation for the *Hemistola chrysoprasaria* assembly (GCA_947063395.1) in Ensembl Rapid Release.

### Wellcome Sanger Institute – Legal and Governance

The materials that have contributed to this genome note have been supplied by a Darwin Tree of Life Partner. The submission of materials by a Darwin Tree of Life Partner is subject to the **‘Darwin Tree of Life Project Sampling Code of Practice’**, which can be found in full on the Darwin Tree of Life website here. By agreeing with and signing up to the Sampling Code of Practice, the Darwin Tree of Life Partner agrees they will meet the legal and ethical requirements and standards set out within this document in respect of all samples acquired for, and supplied to, the Darwin Tree of Life Project.

Further, the Wellcome Sanger Institute employs a process whereby due diligence is carried out proportionate to the nature of the materials themselves, and the circumstances under which they have been/are to be collected and provided for use. The purpose of this is to address and mitigate any potential legal and/or ethical implications of receipt and use of the materials as part of the research project, and to ensure that in doing so we align with best practice wherever possible. The overarching areas of consideration are:

- Ethical review of provenance and sourcing of the material
- Legality of collection, transfer and use (national and international)

Each transfer of samples is further undertaken according to a Research Collaboration Agreement or Material Transfer Agreement entered into by the Darwin Tree of Life Partner, Genome Research Limited (operating as the Wellcome Sanger Institute), and in some circumstances other Darwin Tree of Life collaborators.

## Data Availability

European Nucleotide Archive: *Hemistola chrysoprasaria.* Accession number PRJEB55573; https://identifiers.org/ena.embl/PRJEB55573. ( Wellcome Sanger Institute, 2022) The genome sequence is released openly for reuse. The *Hemistola chrysoprasaria* genome sequencing initiative is part of the Darwin Tree of Life (DToL) project. All raw sequence data and the assembly have been deposited in INSDC databases. Raw data and assembly accession identifiers are reported in Table 1.

## Author information

Members of the University of Oxford and Wytham Woods Genome Acquisition Lab are listed here: https://doi.org/10.5281/zenodo.4789928.

Members of the Darwin Tree of Life Barcoding collective are listed here: https://doi.org/10.5281/zenodo.4893703.

Members of the Wellcome Sanger Institute Tree of Life programme are listed here: https://doi.org/10.5281/zenodo.4783585.

Members of Wellcome Sanger Institute Scientific Operations: DNA Pipelines collective are listed here: https://doi.org/10.5281/zenodo.4790455.

Members of the Tree of Life Core Informatics collective are listed here: https://doi.org/10.5281/zenodo.5013541.

Members of the Darwin Tree of Life Consortium are listed here: https://doi.org/10.5281/zenodo.4783558.
