# Supplementary material for: The genome sequence of the Small Emerald, Hemistola chrysoprasaria (Esper, 1795)
Source: Wellcome Open Res. 2023 Oct 12;8:441. [Version 1] doi: 10.12688/wellcomeopenres.19999.1 (PMC11367073; doi:10.12688/wellcomeopenres.19999.1)
Supplement: Peer review history

# Reviewer report: Reviewer response for version 1

**DOI:** 10.21956/wellcomeopenres.22145.r93440

**Published:** 2024-09-01

**Authors:** Fathiya Khamis, Inusa Ajene

**Affiliations:** 1 International Centre of Insect Physiology and Ecology Nairobi, Nairobi, Kenya

The data note titled “The genome sequence of the Small Emerald, *Hemistola chrysoprasaria* (Esper, 1795)” presents the genome assembly of the Small Emerald ( *Hemistola chrysoprasaria)* moth. The paper is thorough, describing the background of the insect, the genome assembly and annotation. The methods are clearly described, and the protocols used are current and technically sound with publicly available references to databases and software. This is a good paper with impact as a potential resource for future genomic studies on the biology of the moth and the molecular basis of the colour change behaviour in the larvae.

Are sufficient details of methods and materials provided to allow replication by others?

Yes

Is the rationale for creating the dataset(s) clearly described?

Yes

Are the datasets clearly presented in a useable and accessible format?

Yes

Are the protocols appropriate and is the work technically sound?

Yes

Reviewer Expertise:

Molecular Biologist

We confirm that we have read this submission and believe that we have an appropriate level of expertise to confirm that it is of an acceptable scientific standard.

# Reviewer report: Reviewer response for version 1

**DOI:** 10.21956/wellcomeopenres.22145.r89120

**Published:** 2024-08-06

**Authors:** Chuanlin Yin

**Affiliations:** 1 China Jiliang University, Hangzhou, China

The authors conducted a thorough genome sequencing of the small emerald, Hemistola chrysoprasaria. The results are promising, and the methods are clearly outlined. This paper has reached a publishable level, but there are a few minor issues that should be addressed to further improve it.

**Minor:**

- Table1: Usually, the C+F+M of BUSCO is almost 100%, but this paper is 99.9% (98.2%+0.4%+1.3%), please check the data.
-  Genome annotation: the BRAKER2 pipeline was used in default protein mode to generate annotation, what the homology protein sets did the authors used, need to specify.
-  Table 3: the braker software is missing.

Are sufficient details of methods and materials provided to allow replication by others?

Yes

Is the rationale for creating the dataset(s) clearly described?

Yes

Are the datasets clearly presented in a useable and accessible format?

Yes

Are the protocols appropriate and is the work technically sound?

Yes

Reviewer Expertise:

Insectomics and bioinformatics

I confirm that I have read this submission and believe that I have an appropriate level of expertise to confirm that it is of an acceptable scientific standard.
